# Neuroinflammation Induced by Transgenic Expression of Lipocalin-2 in Astrocytes

**DOI:** 10.3389/fncel.2022.839118

**Published:** 2022-02-23

**Authors:** Jae-Hong Kim, Osung Kwon, Anup Bhusal, Jiyoun Lee, Eun Mi Hwang, Hoon Ryu, Jae-Yong Park, Kyoungho Suk

**Affiliations:** ^1^Department of Pharmacology and Department of Biomedical Science, School of Medicine, Kyungpook National University, Daegu, South Korea; ^2^Brain Science & Engineering Institute, Kyungpook National University, Daegu, South Korea; ^3^Department of Integrated Biomedical and Life Science, Graduate School, Korea University, Seoul, South Korea; ^4^BK21FOUR R&E Center for Learning Health Systems, Korea University, Seoul, South Korea; ^5^Center for Functional Connectomics, Brain Science Institute, Korea Institute of Science and Technology, Seoul, South Korea; ^6^Center for Neuroscience, Brain Science Institute, Korea Institute of Science and Technology, Seoul, South Korea; ^7^Veterans Affairs Boston Healthcare System, Boston, MA, United States; ^8^Boston University Alzheimer’s Disease Center and Department of Neurology, Boston University School of Medicine, Boston, MA, United States

**Keywords:** transgenic mice, astrocytes, lipocalin-2, neuroinflammation, hippocampus

## Abstract

Transgenic mice are a useful tool for exploring various aspects of gene function. A key element of this approach is the targeted overexpression of specific genes in cells or tissues. Herein, we report for the first time, the generation and characterization of conditional transgenic (cTg) mice for lipocalin-2 (LCN2) expression. We generated the R26-LCN2-transgenic (LCN2-cTg) mice that carried a loxP-flanked STOP (neo) cassette, *Lcn2* cDNA, and a GFP sequence. When bred with Tg mice expressing Cre recombinase under the control of various tissues or cell-specific promoters, Cre-mediated recombination deletes the STOP cassette and allows the expression of LCN2 and GFP. In this study, we achieved the recombination of loxP-flanked LCN2 in hippocampal astrocytes of cTg mouse brain, using a targeted delivery of adeno-associated virus (AAVs) bearing Cre recombinase under the control of a GFAP promoter (AAVs-GFAP-mCherry-Cre). These mice with localized LCN2 overexpression in astrocytes of the hippocampus developed neuroinflammation with enhanced glial activation and increased mRNA and protein levels of proinflammatory cytokines. Furthermore, mice showed impairment in cognitive functions as a typical symptom of hippocampal inflammation. Taken together, our study demonstrates the usefulness of LCN2-cTg mice in targeting specific cells at various organs for conditional LCN2 expression and for subsequent investigation of the functional role of cell-type-specific LCN2 within these sites. Moreover, the LCN2-cTg mice with targeted expression of LCN2 in hippocampal astrocytes are a new *in vivo* model of neuroinflammation.

## Introduction

Mouse models have been instrumental in expanding our knowledge of various biological mechanisms. In particular, transgenic mouse models having inducible and cell-type restricted expression systems are increasingly used to investigate the molecular mechanisms by which gene products influence cellular processes (Leung and Jia, 2016). Lipocalin-2 (LCN2) is an acute-phase protein produced in response to injury, infection, or other inflammatory conditions (Suk, 2016; Bhusal et al., 2019b). In the brain, it is highly expressed in glial cells, neurons, and endothelial cells, where it regulates neuroinflammation (Jha et al., 2015). LCN2 has been identified as a modulator of various cellular phenotypes, including cell morphology, migration, functional polarization, differentiation, and survival or death. However, most of these findings were based on the loss-of-function approach using *Lcn2*-knockout (KO) mice. These strategies have limitations, as related family members can mask phenotypes and compensate for the KO of a gene from the developmental stage of animals (Barbaric et al., 2007). Thus, a gain-of-function approach overcomes these problems and has proven to be a useful strategy for analyzing gene function in many model organisms (Prelich, 2012).

Herein, we report the generation and characterization of the LCN2 conditional transgenic (LCN2-cTg) mice. These mice can be used to overexpress LCN2 in a Cre-inducible and cell/tissue-specific manner. Moreover, recombinant adeno-associated virus (AAV) vectors are a popular genetic approach to confer an efficient transgene expression in a cell/tissue-specific manner (Aponte-Ubillus et al., 2018; Wang et al., 2019). In this study, we used AAVs bearing Cre recombinase under the GFAP promoter (AAVs-GFAP-mCherry-Cre) to enable the transgene (LCN2) expression in hippocampal astrocytes of cTg mice. We then analyzed hippocampal inflammatory phenotypes and consequent behavioral changes in these mice. Our data supported the use of this novel LCN2-cTg mice as a platform for studying cell/tissue-specific roles of LCN2 and related signaling pathways.

## Materials and Methods

### Animals

The C57BL/6 mice were provided by Samtako Bio Korea. All animal experiments were carried out on adult mice aged 8–12 weeks (both male and female). Mice were housed under standard conditions of light/dark cycle (12 h). Experiments were performed as per the protocols approved by Korea University Institutional Animal Care and Use Committee and Kyungpook National University (KUIACUC-2019-0050 and KNU 2019-09, respectively).

### Neuroinflammation Model Based on Intraperitoneal LPS Injections

We injected 5 mg/kg of LPS (Sigma-Aldrich, St Louis, MO) through the intraperitoneal route to evoke neuroinflammation as previously described (Qin et al., 2007). The same amount of saline was given to the animals in the vehicle control group. The animals were then given a deep urethane-induced anesthesia and sacrificed after 24 h.

### Generation and Maintenance of LCN2-cTg Mice

gBlocks Gene Fragments (Integrated DNA Technologies, Coralville, IA) constructed FseI-LCN2-2A-GFP-FseI, which was inserted into the FseI site of the Ai6 plasmid vector (Addgene, Cat#: 22798, Watertown, MA) to generate conditional LCN2 overexpression transgene constructs (Feng et al., 2017). Subsequently, the LCN2 transgene construct was digested with AvrII/SacI and micro-injected into C57BL/6N-derived embryonic stem cells. Whole-genome sequencing was conducted using the Miseq system (Illumina, San Francisco, CA) to determine the location of the inserted transgenic genes in the Rosa26 locus. Afterward, chimeric mice were maintained by breeding with C57BL/6N mice for three-four generations. LCN2-cTg mice were finally genotyped using a pair of primers as follows: 5′-GAG CTA CAA TGT GCA AGT GGC-3′ (forward), 5′-GAA CTT GTG GCC GTT TAC GTC-3′ (reverse).

### Cell Culture and Transfection

We cultured primary astrocytes from the hippocampus of mouse pups (P1) as described previously (Lee et al., 2007). Briefly, day-old LCN2-cTg mice hippocampus was chopped and mechanically disrupted through trituration. The astrocytes were then seeded in 75 cm^2^ culture plates and grown at 37°C in a 5% CO_2_ atmosphere in DMEM with 10% FBS and 1% penicillin-streptomycin. After 3–4 days, the culture medium was changed to eliminate debris and other floating cell types. Subsequently, cells were used for transfection after 5–7 days of culture.

For transient transfection, cells were treated with pDEST-mCherry or pDEST-mCherry-Cre cDNA using a Neon Electroporation Device (ThermoFisher, Waltham, MA), following an optimized voltage protocol. The transient transfectants were then identified based on their mCherry expressions as observed under a Ti2 confocal microscope (Nikon, RRID: SCR_021068, Tokyo, Japan). These transfected cells were then subjected to immunocytochemical staining and Western blotting and astrocyte-conditioned medium (ACM) was used for ELISA.

### Immunofluorescence Staining

The immunofluorescence staining of transfected cells and animal tissues was performed as previously described (Kim et al., 2017). For this, cells and brain tissue slices were incubated with mouse anti-s100β (Sigma-Aldrich, St. Louis, MO), mouse anti-GFAP (BD Biosciences, San Diego, CA), rabbit anti-GFAP (Dako, Glostrup, Denmark), rabbit anti-Iba-1 (Wako, Osaka, Japan), goat anti-Iba-1 (Novus Biologicals, Centennial, CO), rabbit anti-NeuN (Millipore, Burlington, MA), and goat anti-LCN2 (R&D systems, Minneapolis, MN) antibodies. Following incubation with the primary antibodies, tissue sections were rinsed and incubated with secondary antibodies (FITC-, Cy5-, Cy3-, and Brilliant violet-conjugated; The Jackson Laboratory, Bar Harbor, ME). Slides were washed and then cover-slipped with a mounting medium containing DAPI (Vector Laboratories, Burlingame, CA). The images of tissue sections were taken under a confocal microscope. The immunoreactivity was then analyzed using ImageJ software (NIH, Bethesda, MD). For quantification of cells number, we set a GFAP or Iba-1 secondary mask within the DAPI primary mask and counted the cells with double positive cells using Gen 5 software (BioTek, Winooski, VT).

Analysis of microglia morphology was conducted as described previously (Zhao et al., 2018). In brief, confocal image stacks were collected using a 4× objective lens with a 0.5-μm interval through a 10-μm z-depth of the tissue. The image stacks were processed by maximum intensity projection to create 2D images. The 2D images were then imported into the Zen blue lite 2.3 program (Carl Zeiss, Oberkochen, Germany). Cell body sizes were measured using “Draw Spline Contour” in the “Graphics” section. Microglia were divided into two groups: ramified cells were regarded as resting microglia, while spherical cells with fewer branches were considered activated. A cell-body diameter of 10 μm was set as the cut-off criterion. Microglia possessing smaller cell bodies with long, lean, and relatively more branches were considered to be resting microglia. In contrast, microglia with a cell body larger than 10 μm with thick and hardly any branches were regarded as activated.

### Western Blotting

The concentration of protein extracted from transfected cells was measured using a bicinchoninic acid assay (Pierce, Waltham, MA; Kim et al., 2021b). Protein samples were separated on SDS-PAGE gels. The separated protein samples were then transferred to PVDF membranes (Bio-Rad, Hercules, CA) and incubated with 5% skim milk for 30 min. The membranes were washed and treated with primary antibodies against goat anti-LCN2 (R&D systems, Minneapolis, MN), mouse anti-GFP (Santa Cruz Biotechnology, Dallas, TX), rabbit anti-α-tubulin (Cell Signaling Technology, Denver, CO). The next day, the membranes were washed and incubated with secondary antibodies (Jackson ImmunoResearch, Bar Harbor, ME), followed by enhanced chemiluminescence detection (Thermo Fisher Scientific, Waltham, MA).

### ELISA

LCN2, IL-1β, and TNF-α levels in the ACM and extracellular fluid (ECF) were quantified using mouse LCN2, TNF-α, and IL-1β ELISA kits (R&D Systems, Minneapolis, MN). The assays were carried out in 96-well plates as per the manufacturer’s instructions. For standards, mouse recombinant LCN2, IL-1β, and TNF-α were used at concentrations ranging from 0 to 2,500 pg/ml. All measurements were obtained from duplicate assays.

### Viral Gene Transfers *In vivo*

AAV5-GFAP-mCherry and AAV5-GFAP-mCherry-Cre (University of North Carolina Vector Core; 4.3 × 10^9^ virus particles per side, Chapel Hill, NC) viral constructs were used in the study. To inject the virus, mice were maintained under isoflurane anesthesia and located in a stereotaxic device. The injection needles were placed bilaterally on the hippocampal CA1 using the following coordinates: AP −2 mm, L ± 1.8 mm from the bregma, and V −1.2 mm from the dura (Kim et al., 2021b). Then, the virus was delivered (total; 0.5 μl, rate; 0.1 μl/min) into the hippocampus. The needle of injection was removed after 10 min. Mice were provided with immediate postoperative care and we waited for 14 days for them to fully recover before the start of any new experiment. The astrocyte-specific expression of mCherry-Cre was confirmed through immunofluorescence staining.

### Microdialysis

Microdialysis was conducted as previously described with slight modifications (Kim et al., 2017). Mice were maintained under isoflurane anesthesia and placed on a stereotaxic device. Following that, the guide cannula was inserted into the hippocampal CA1 region. For this, the guide cannula tips were lowered to 0.5 mm above the target: AP −2 mm, L ± 1.8 mm from the bregma, and V −1.2 mm from the dura. The dental cement and three anchor screws were used to fix the guide cannula in the mouse skull. Mice were housed individually and allowed to recover for 7 days after the cannula implantation. Then, the microdialysis probe (CMA Microdialysis AB, Kista, Sweden) was inserted into the CA1 region, using a guide cannula. The probe was connected to a microperfusion pump with polyethylene tubing and perfused with artificial CSF (aCSF) at a flow rate of 0.5 μl/min. ECF from the outlet and of the tube was collected in plastic vials kept on ice. Samples were collected for 2 h daily and immediately frozen at −80°C to use later for ELISA.

### Quantitative Real-Time Polymerase Chain Reaction (qPCR)

QIAzol reagent (QIAGEN, Hilden, Germany) was used to extract total RNA from hippocampal tissues or cells according to the manufacturer’s instructions. The qPCR assay was then performed according to the manufacturer’s instructions using the one-step SYBR PrimeScript^TM^ RT-PCR kit (Takara, Tokyo, Japan), followed by detection using the ABI Prism^®^ 7000 sequence detection system (Applied Biosystems, Foster City, CA). The 2^−ΔΔCT^ method was used to calculate relative changes in gene expression determined through qPCR experiments (Livak and Schmittgen, 2001). Primers used in qPCR analyses were as follows: *Lcn2*, 5′-ATG TCA CCT CCA TCC TGG TC-3′ (forward), 5′-CAC ACT CAC CAC CCA TTC AG-3′ (reverse); *Tnf*, 5′-CAT CTT CTC AAA ATT CGA GTG ACA A-3′ (forward), 5′-ACT TGG GCA GAT TGA CCT CAG-3′ (reverse); *Il1b*, 5′-AGT TGC CTT CTT GGG ACT GA-3′ (forward), 5′-TCC ACG ATT TCC CAG AGA AC-3′ (reverse); and *Gapdh*, 5′-TGG GCT ACA CTG AGG ACC AG-3′ (forward), 5′-GGG AGT TGC TGT TGA AGT CG-3′ (reverse).

### Behavioral Testing

To assess different aspects of hippocampal function, we used three different behavioral tests: the Y-maze test, novel object recognition test, and passive avoidance test. At the start of each experiment, mice were habituated to the testing room for 30 min. The same experimenter conducted all of the tests, and each mouse was tested in the same order as the others. The experimenter was separated from the testing area during the trials, and the test trails were recorded, tracked, and analyzed using Smart v3.0 software (Panlab, Barcelona, Spain). During behavioral tests, the experimenter was blinded to the genotype and treatment conditions of the animals, who were only identified by their subject number.

#### The Y-Maze Test

The spatial cognition of experimental animals was determined by measuring the spontaneous alteration in the Y-maze with slight modifications (Kim et al., 2017). Mice were placed within the center of Y-maze, after which the sequence and number of arm entries were recorded manually for each animal over a 7-min period. A spontaneous alternation was defined as entries into all three arms on consecutive choices. The total number of arm entries indicated the locomotor activity of animals. The maze arms were thoroughly cleaned between animal experiments to remove any residual odors. The percentage of alternations and the total number of arm entries was measured using a previously established formula.

#### The Novel-Object Recognition Test (NOR)

The NOR test was conducted as per the previously established protocol (Kim et al., 2017). Animals were first exposed to the testing arena for 5 min before being introduced to two identical objects for 10 min as a part of their training session. In the test session, mice were placed in the same arena with one of the familiar objects being replaced with the newer one. The velocity, exploration, and total distance covered by mice were recorded for 10 min and analyzed using a previously established formula.

#### The Passive Avoidance Test

All recording and operation of the passive avoidance test were conducted using the manufacturer’s software (Gemini Systems, Lincoln, UK) as described previously (Kim et al., 2021a). In the training session, the animals were placed in the light compartment which when moved to the dark compartment received a mild electric shock (0.25 mA/1 s. The latency (s) to enter the dark chamber during the training session was taken as baseline values. After 24 h, in the probe test session, the mouse latency to move to the dark compartment was measured as an index of passive avoidance.

### Statistical Analysis

All data were presented as mean ± SEM and different statistical tests were performed (see figure legends) using the SPSS software (version 18.0; SPSS Inc., Chicago, IL). Statistical significance was set at *p* < 0.05. The sample size for the experiments was chosen to ensure adequate statistical power based on the G*power 3.1 software (Heinrich-Heine-Universität Düsseldorf, Düsseldorf, Germany; Faul et al., 2007).

## Results

### Generation of LCN2 Conditional Transgenic Mice

**Figure 1:**
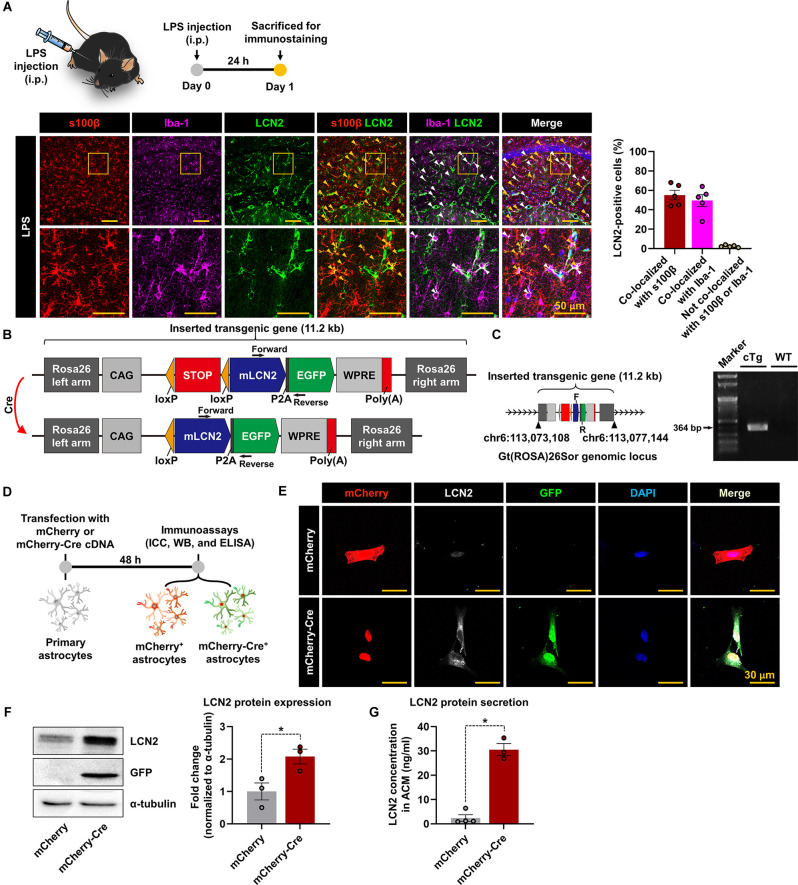
The generation of LCN2-cTg mice. **(A)** A schematic showing the experimental timelines. LPS-injected brain sections containing the hippocampal region were subjected to immunofluorescence analysis to assess the expression of LCN2 (green) in s100β-positive (red, an astrocytes marker) or Iba-1-positive (magenta, a microglial marker) cells. LCN2 expression was co-localized with either astrocytes (yellow arrowheads), microglia (white arrowheads), and nuclei (blue). The quantification graph is presented as means ± SEM (*n* = 5, *right*). **(B)** Schematic design of LCN2-cTg mice. Inserted transgenic genes were designed to express mouse LCN2 (mLCN2) and EGFP with the CAG promoter only in Cre-expressing cells. **(C)** Whole-genome sequencing (WGS) results, indicating the location of the inserted transgenic gene in the Rosa26 locus (*left*); F, forward; R, reverse. Genotype PCR results with designed primer pair (*right*). **(D)** Diagram showing the timeline of experimentation. Cultured astrocytes were transfected with a mCherry or mCherry-Cre expression construct. After 48 h, immunocytochemistry (ICC) **(E)**, Western blot (WB) **(F)**, and ELISA **(G)** of transfected cells or culture media were conducted. **(E)** Microscopic data showing the colocalization of mCherry, LCN2, and GFP in mCherry-Cre-transfected astrocytes. **(F)** Protein levels of LCN2 and GFP, assessed by Western blotting in transfected astrocytes (*n* = 3). **(G)** The extracellular LCN2 protein concentration was measured from the astrocyte-conditioned medium (ACM, *n* = 3–4). Data are presented as the means ± SEM (*p < 0.05, between the indicated groups).

We first used an LPS-induced neuroinflammation model to examine the cellular source of LCN2 in the inflamed brain. Immunofluorescence staining revealed that LCN2 was primarily expressed in astrocytes (s100β-positive) and microglia (Iba-1-positive) cells in the hippocampus, 24 h after administering the LPS (i.p.; Figure 1A). Based on these results, we decided to generate a new conditional neuroinflammation mouse model by overexpressing LCN2 in glial cells. For this study, we first designed a Cre-dependent LCN2 overexpression construct, with additional GFP expression, after which engineered constructs were used to develop LCN2-cTg mice (Figure 1B). LCN2-cTg mice were then validated through whole-genome sequencing and genotype PCR, using designed primer pairs (Figure 1C). The inserted transgene was observed at the Rosa26 region, where we intended (Figure 1C). Next, to test the efficiency of Cre-mediated recombination, primary astrocytes obtained from transgenic mice were first transfected with mCherry or mCherry-Cre plasmid constructs (Figure 1D). Afterward, the transfection efficiency of plasmid constructs and Cre-mediated recombination processes were confirmed using microscopic evaluation of mCherry-positive cells and LCN2/GFP expression, respectively (Figure 1E). Western blotting and ELISA assays further revealed that mCherry-Cre transfection increased the expression and release of LCN2 protein in astrocytes (Figures 1F,G). Hence, an efficient Cre-dependent expression of LCN2 was triggered in astrocytes, which were isolated from the LCN2-cTg mice.

**Figure 2:**
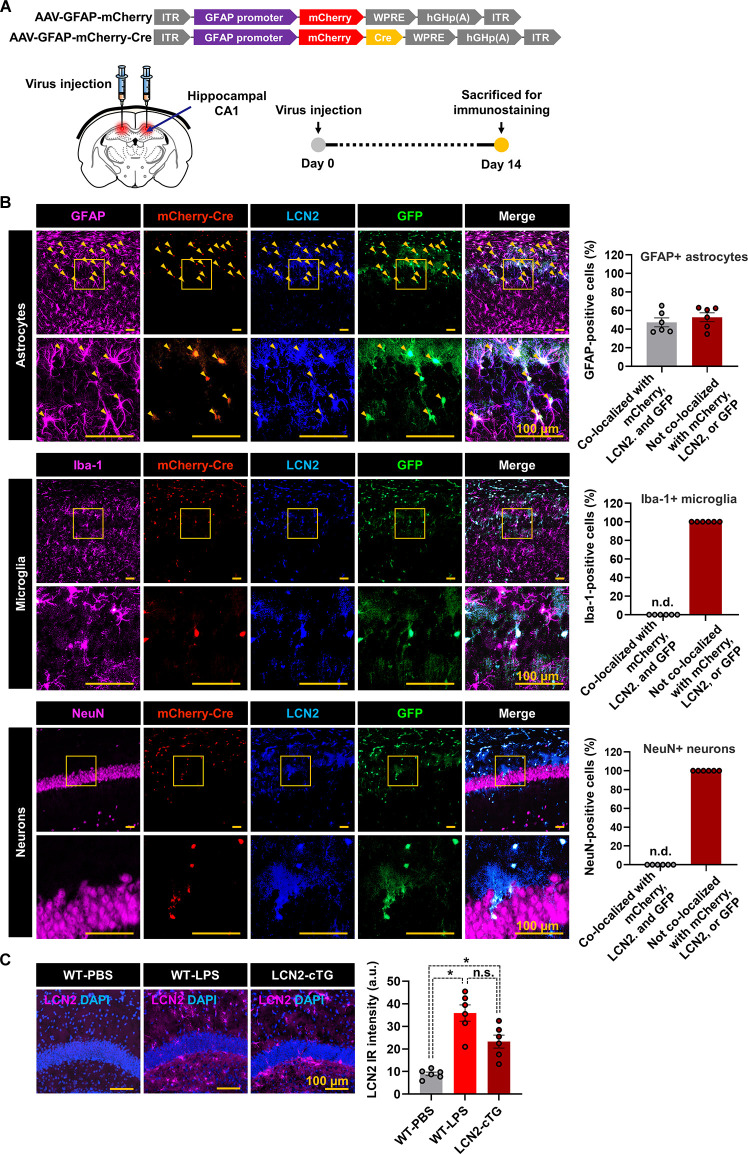
Cre-inducible LCN2 overexpression in hippocampal astrocytes of LCN2-cTg mice. **(A)** An illustration of adeno-associated viral (AAV) vector containing the Cre construct (AAV-GFAP-mCherry and AAV-GFAP-mCherry-Cre). The expression of mCherry or mCherry-Cre is controlled by GfaABC1D, a shortened version of the GFAP promoter (*upper*). A schematic, showing the route of AAV-GFAP-mCherry-Cre administration into the hippocampal CA1 region and experimental timeline (*lower*). **(B)** Following the administration of AAV-GFAP-mCherry-Cre, brain tissue sections were subjected to immunofluorescence analysis to localize the expression of mCherry-Cre (red), LCN2 (blue), and GFP (green) in astrocytes (GFAP, magenta), microglia (Iba-1, magenta), and neurons (NeuN, magenta). Arrowheads (yellow) indicate the colocalization of GFAP, mCherry-Cre, LCN2, and GFP. **(C)** We further compared the LCN2 expression (magenta) in the hippocampal region of LPS-injected and LCN2-cTg mice (*n* = 6). DAPI for nuclei (blue). The quantification of colocalization is shown in the adjacent graphs (means ± SEM (*p < 0.05, between the indicated groups). n.d., not detected; n.s., not significant; a.u., arbitrary unit.

To further validate the Cre-dependent recombination in astrocytes of LCN2-cTg mice under *in vivo* conditions, we injected AAV-GFAP-mCherry-Cre into the hippocampus (Figure 2A). Immunofluorescence staining revealed the cellular localization of mCherry-Cre in GFAP-positive astrocytes in the hippocampus, 14 days after viral injection (Figure 2B). No considerable immunoreactivity for mCherry-Cre was observed in the microglia or neurons (NeuN-positive) in the hippocampus. The expression of LCN2 and GFP was also restricted to hippocampal astrocytes, suggesting the successful and specific Cre-mediated recombination occurring in astrocytes. Our immunofluorescence analysis further revealed that LCN2 expression level in the hippocampus of LCN2-cTg mice was similar to that of the established LPS-induced neuroinflammatory model (Figure 2C).

### Astrocyte-Specific Transgenic Expression of LCN2 Induces Neuroinflammation

**Figure 3:**
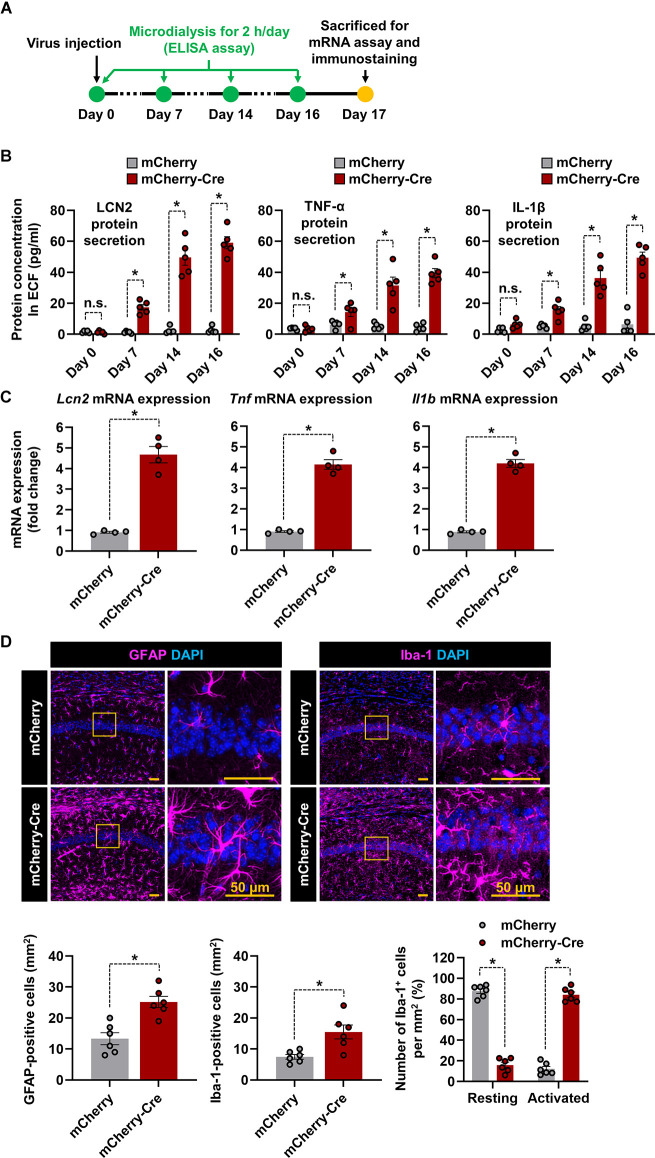
Overexpression of LCN2 in the hippocampal astrocytes induces neuroinflammation. **(A)** The experimental timeline. **(B)** Following administration of either AAV-GFAP-mCherry or AAV-GFAP-mCherry-Cre, LCN2, IL-1β, and TNF-α levels in microdialysates (*n* = 5) were measured through ELISA. **(C)** The hippocampal tissues were collected from each mouse and obtained total RNA was subjected to qPCR to determine expression levels of *Lcn2*, *Tnf*, and *Il1b* mRNA normalized by *Gapdh*. **(D)** The hippocampal CA1 region was used for quantification of astrocytes (magenta) or microglia (magenta) colocalized with DAPI (blue). The percentage of microglia displaying a resting and activated morphology in the hippocampal CA1 region (*n* = 6). Data are presented as means ± SEM (*p < 0.05, between the indicated groups); n.s., not significant.

We investigated whether astrocyte-specific LCN2 overexpression influences the production of proinflammatory cytokines and glial activation in LCN2-cTg mice (Figure 3A). For this study, after AAV-GFAP-mCherry-Cre was injected into the hippocampal CA1 region, extracellular fluid (ECF) was obtained at 0, 7, 14, and 16 days using a microdialysis probe. We observed significantly elevated levels of LCN2 and proinflammatory cytokines in the dialysate after the AAV-GFAP-mCherry-Cre injection (Figure 3B). qPCR results further showed significant induction of *Lcn2*, *Tnf*, and *Il1b* mRNA expression in the hippocampal tissue of LCN2-cTg mice (Figure 3C). Next, we immunostained brain sections at 17 d after viral injection. As shown in Figure 3D, we observed an increase in the number of microglia and astrocytes in the hippocampal CA1 region. Microglia also showed morphological changes from resting to activated states in the hippocampus of LCN2-cTg mice (Figure 3D, *right*). These data, therefore, propose that astrocyte-specific LCN2 overexpression in the hippocampus causes neuroinflammation.

### Astrocyte-Specific Transgenic Expression of LCN2 in the Hippocampus Elicits Cognitive Deficits

**Figure 4:**
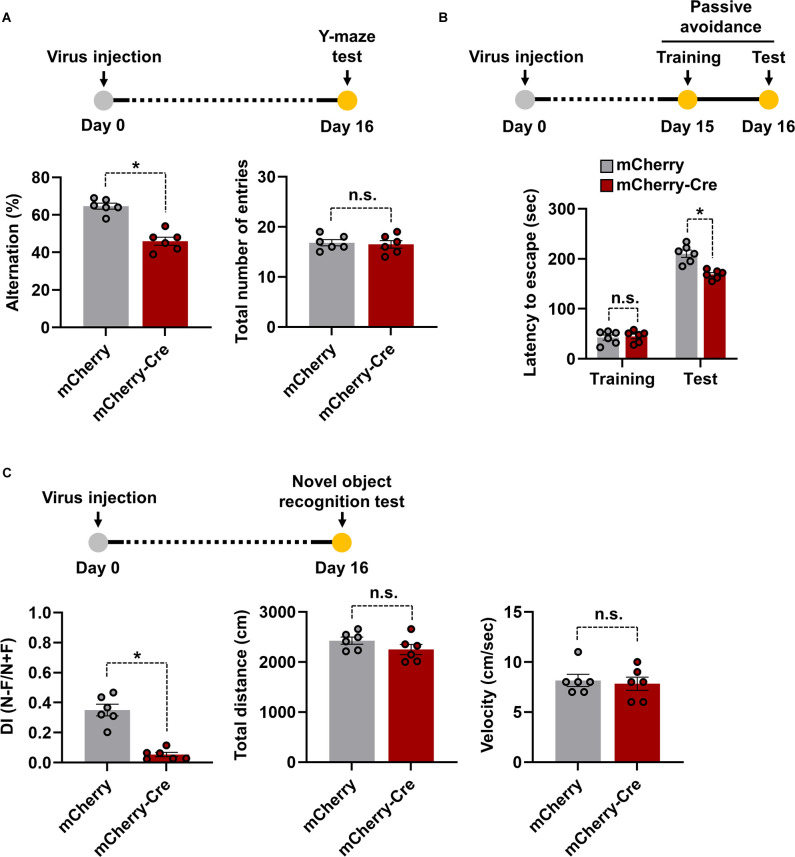
Effects of astrocyte-specific cre-mediated LCN2 overexpression in the hippocampus on learning and memory functions. LCN2-cTg mice were injected with AAV-GFAP-mCherry or AAV-GFAP-mCherry-Cre in the hippocampus. Cognitive behavior of animals was then analyzed using a Y-maze (*n* = 6) **(A)**, passive avoidance (*n* = 6) **(B)**, and novel object recognition (*n* = 6) **(C)** tests. Experimental timelines of viral injection and behavioral analysis are also shown (*upper*). Data are presented as means ± SEM (*p < 0.05, between the indicated groups); n.s., not significant.

Hippocampus is one of the important brain regions which is involved in learning and memory function (Anand and Dhikav, 2012). Several studies have found that any injury or inflammation to the hippocampus causes learning deficits, cognitive and mood dysfunction, and related behavioral responses (de Haan et al., 2006; Dinel et al., 2011). Based on this idea, we investigated whether overexpression of LCN2 in cTg mice hippocampal astrocytes resulted in similar phenotypes. For this, AAV-GFAP-mCherry-Cre-injected LCN2-cTg mice were subjected to several learning and memory function tests. The spontaneous alternation measurement using a Y-maze was used as a test for habituation and spatial working memory (Wolf et al., 2016). A mouse that shows no preference for a new environment (arm) during the test is an indication of impaired spatial memory, which may indicate impaired functioning of the hippocampus (Kraeuter et al., 2019). We observed a significant decrease in spontaneous alternation in the mCherry-Cre virus-injected transgenic mouse group compared to the mCherry control virus-injected one. No significant difference in total arm entries was observed between the groups (Figure 4A). The hippocampus is also known for processing contextual information, necessary for contextual fear acquisition and its extinction. The hippocampal disorder enhances the development of persistent stress and, thus, may confer vulnerability to the development of anxiety disorders (Cominski et al., 2014). With the passive avoidance test, after 24 h of mild electric shock exposure, we observed a significant decrease in latency to enter the dark compartment in mCherry-Cre virus-injected transgenic mice when compared to mCherry control virus-injected animals (Figure 4B). Alternatively, the novel object recognition test is used to evaluate recognition memory in mice and is based on the natural tendency of rodents to explore a novel object longer than a familiar one (Cohen and Stackman, 2015). LCN2-cTg mice injected with mCherry-Cre virus showed a lower tendency to interact with novel objects when compared to mCherry control virus-injected transgenic animals (Figure 4C). No significant difference in the total distance covered and velocity was observed between the groups. These findings, therefore, indicate that LCN2-cTg mice with astrocyte-specific transgenic expression of LCN2 in the hippocampus show learning and memory dysfunction.

## Discussion

In this study, we generated a novel conditional transgenic mouse line, which expressed the LCN2 transgene in a Cre recombinase-dependent manner. Also, the expression of the transgene was driven by the CAG promoter. The CAG promoter used here offers a significant advantage, as it provides a more stable and ubiquitous expression of the transgene in offsprings (Sakai and Miyazaki, 1997). This provision allows LCN2-cTg mice to be used in a broader range of studies by exploiting various cell-specific Cre vectors or Cre mouse lines. Moreover, this system has the significant advantage of GFP co-expression, thus allowing convenient monitoring of LCN2 expression in different tissues/cells. In this study, we showed the selective expression of LCN2 and GFP transgenes in astrocytes using the mCherry-Cre construct or AAV-GFAP-mCherry-Cre under *in vitro* and *in vivo* conditions, respectively. Transfection of cultured astrocytes with the mCherry-Cre construct, or the delivery of AAV-GFAP-mCherry-Cre into the hippocampus of LCN2-cTg mice, led to the astrocyte-specific recombination, thereby resulting in efficient transgene expression.

LCN2 expression in astrocytes has been observed to regulate neuroinflammation under various brain injury conditions (Suk, 2016). In this study, the Cre-mediated LCN2 overexpression in astrocytes induced neuroinflammation, with increased gliosis and inflammatory cytokines being observed in the hippocampus of LCN2-cTg mice. Behavioral experiments further revealed that the elevated astrocyte LCN2 in the hippocampus impaired learning and memory function in the LCN2-cTg mice. Previously, individuals with cognitive impairment showed an elevated level of LCN2 in the plasma (Choi et al., 2011). Similarly, a recent study showed that rodents receiving exogenous LCN2 displayed hippocampal neuronal dysfunction and memory impairment (Olson et al., 2021). Our study using LCN2-cTg mice and the hippocampus-targeted delivery of AAV-GFAP-mCherry-Cre further demonstrated the precise effects of astrocyte LCN2 on hippocampal neuroinflammation and consequent behaviors. While research into the precise mechanisms underlying these phenotypes is underway, we can assume that LCN2 overproduced in the hippocampus of LCN2-cTg mice binds to its receptors like 24p3R, which is present throughout the brain in neurons and glial cells (Ip et al., 2011; Jin et al., 2014; Suk, 2016; Zhao et al., 2019). Further, LCN2 has been reported to induce the expression of its receptors on its own in the brain cells (Mondal et al., 2020). Once bound to glial cells, LCN2 activates various inflammatory pathways in glial cells causing gliosis, cytokines production, and augmentation of an inflammatory response (Jha et al., 2015). LCN2, on the other hand, is an iron trafficking protein that has been linked to sequestering intracellular iron and suppressing iron-responsive genes in different cell types. In the hippocampus, it has been shown to regulate neuronal spine density, neurogenesis, and neuronal dysfunction in both physiological and disease condition (Mucha et al., 2011; Ferreira et al., 2013; Skrzypiec et al., 2013; Kim et al., 2017; Bhusal et al., 2019a; Olson et al., 2021). Taken together, we report the development of LCN2-cTg mice that can be used to overexpress LCN2 in a cell/tissue-specific manner. These transgenic mice can be used in future LCN2 studies, and represent a new platform for the use of animal models in studying neuroinflammation and peripheral inflammation.

## Data Availability Statement

The datasets presented in this study can be found in online repositories. The names of the repository/repositories and accession number(s) can be found in NCBI SRA SRR17332112.

## Ethics Statement

The animal study was reviewed and approved by Korea University Institutional Animal Care & Use Committee (KUIACUC-2019-0050) and Kyungpook National University (KNU 2019-09).

## Author Contributions

All authors have made a substantial intellectual contribution to this work and have approved the submission of the manuscript.

## Conflict of Interest

The authors declare that the research was conducted in the absence of any commercial or financial relationships that could be construed as a potential conflict of interest.

## Publisher’s Note

All claims expressed in this article are solely those of the authors and do not necessarily represent those of their affiliated organizations, or those of the publisher, the editors and the reviewers. Any product that may be evaluated in this article, or claim that may be made by its manufacturer, is not guaranteed or endorsed by the publisher.
